# Flavonoid and Phenolic Acids Content and In Vitro Study of the Potential Anti-Aging Properties of *Eutrema japonicum* (Miq.) Koidz Cultivated in Wasabi Farm Poland

**DOI:** 10.3390/ijms22126219

**Published:** 2021-06-09

**Authors:** Katarzyna Szewczyk, Wioleta Pietrzak, Katarzyna Klimek, Małgorzata Miazga-Karska, Agnieszka Firlej, Marek Flisiński, Anna Grzywa-Celińska

**Affiliations:** 1 Department of Pharmaceutical Botany, Medical University of Lublin, 1 Chodźki Str., 20-093 Lublin, Poland; wioleta.pietrzak@umlub.pl; 2 Department of Biochemistry and Biotechnology, Medical University of Lublin, 1 Chodźki Str., 20-093 Lublin, Poland; katarzyna.klimek@umlub.pl (K.K.); malgorzata.miazga-karska@umlub.pl (M.M.-K.); 3 Wasabi Farm Poland, 5B Słoneczna Str. 26-630 Jedlnia-Letnisko, Poland; sklep@wasabi-farm.eu (A.F.); marek.flisinski@gmail.com (M.F.); 4 Chair and Department of Pneumonology, Oncology and Allergology, Medical University of Lublin, 8 Jaczewskiego Str., 20-090 Lublin, Poland; annagrzywacelinska@umlub.pl

**Keywords:** flavonoids, phenolic acids, *Eutrema japonicum*, *Brassicaceae*, antiaging activity

## Abstract

Skin aging is a natural, unavoidable, and complex process caused by oxidative stress. As a consequence, it leads to an increase in the activation of extracellular matrix disruption enzymes and DNA damage. The search for natural sources that inhibit these mechanisms can be a good approach to prevent skin aging. The purpose of our study was to evaluate the composition of flavonoids and phenolic acids in the extracts obtained from the flowers, roots, and leaves of *Eutrema japonicum* cultivated in Poland. Then, the resultant extracts were subjected to an assessment of antioxidant, anti-collagenase, anti-elastase, anti-hyaluronidase, antibacterial, and cytotoxic properties. It was demonstrated that the extract from the flowers had the highest content of flavonoid glycosides (17.15 mg/g DE). This extract showed the greatest antioxidant, anti-collagenase, anti-elastase, and anti-hyaluronidase activities compared to the other samples. Importantly, the collagenase inhibitory activity of this extract (93.34% ± 0.77%) was better than that of positive control epigallocatechin gallate (88.49% ± 0.45%). An undeniable advantage of this extract was also to possess moderate antibacterial properties and no cytotoxicity towards normal human skin fibroblasts. Our results suggest that extracts from *E. japonicum* flowers may be considered as a promising antiaging compound for applications in cosmetic formulations.

## 1. Introduction

The plant kingdom is a valuable source of polyphenols, such as flavonoids, which exhibit beneficial antioxidant, antibacterial, antiaging, antiviral, antifungal, and anti-inflammatory effects [1]. Thus, the use of plant extracts in cosmetic formulations has been gaining popularity during the past few years. Their use in cosmetic products was found to be more favorable than the application of single biologically active compounds. The search of natural substances with health-promoting activities, such as antioxidant, anti-elastase, and anti-collagenase properties, as well as without cytotoxic effects on the skin, has gained increasing attention. An example is plants rich in polyphenols that have a protective role in many diseases, such as inflammation, cardiovascular illnesses, and cancers. For these reasons, they are used in the healthcare, food, and cosmetics industries [2]. Flavonoids and phenolic acids, in view of their ROS-scavenging activity and/or ability to chelate metals, are often used in cosmetic, especially antiaging, formulations [3]. Currently, various edible plants can be more and more often found in modern cosmetic preparations [1,3].

*Eutrema japonicum* (Miq.) Koidz. (syn. *Wasabia japonica* (Miq.) Matsum., so-called wasabi or Japanese horseradish, belongs to the *Brassicaceae* family [4]. The taxa is mainly cultivated by either a semiaquatic system or a field one. In nature, it grows on the shaded, wet banks of cold mountain springs and streams [5]. The paste of wasabi roots has been used for a long time in Japanese traditional food such as sushi and sashimi as a pungent spice [6]. Literature studies have shown that *E. japonicum* contains not only allyl isothiocyanate derivatives [7,8] but, also, flavonoids [9,10], phenylpropanoids [10,11], and carotenoids [12]. Extracts from the leaves and roots of *E. japonicum* have numerous activities, such as antigenotoxic [13], anti-inflammatory [10,12], antioxidant [10,11], anti-atopic dermatitis [14], antiplatelet [15], anticancer [15,16], and anti-obesity properties [17]. Besides value as a functional and regular food [18,19], the extract of the leaves of wasabi is also used as a cosmetic product, and it is included in the International Nomenclature of Cosmetic Ingredients (INCI) list [20,21].

As a response to the increasing requirements for high-quality and safety cosmetics, there is a growing interest in plant extract products that are rich in many active compounds. In our research, we attempted to demonstrate the benefits arising from the potential use of edible plants, such as *Eutrema japonicum*. As part of the attempt to discover new functional components for antiaging and/or anti-acne preparations, different parts of *E. japonicum* cultivated in Poland were investigated. Therefore, the flavonoid and phenolic acid contents were determined, as well as the antioxidant, anti-collagenase, anti-elastase, antimicrobial, and cytotoxic properties.

## 2. Results and Discussion

### 2.1. Phytochemical Analysis

The total phenolic content (TPC) was determined using Folin–Ciocalteu reagent, and the results were estimated as gallic acid equivalents (GAE) per g of dry extract (DE) (Table 1). Among the four extracts of *E. japonicum*, the extract from the flowers (WJA) had the highest phenolic content (187.79 ± 6.86 mg GAE/g DE), followed by annual leaves (WJC) (77.92 ± 4.82 mg GAE/g DE), biennial leaves (WJD) (54.93 ± 0.99 mg GAE/g DE), and roots (WJB) (31.55 ± 2.17 mg GAE/g DE). It is worth noting that the results obtained for the extracts of *E. japonicum* in our study were better compared to the data presented for the extracts derived from other *Wasabi* species. For instance, Kang et al. [22] demonstrated that TPC for n-hexane and water fractions of 80% ethanol extract of the leaves of *Wasabi koreana* were 13.29 ± 0.43 and 26.35 ± 0.35 mg GAE/g of extract, respectively. Kim et al. [23] found lower amounts of phenolic compounds in the water extracts of the leaves (approx. 0.13 mg GAE/g) and of the roots (approx. 0.04 mg GAE/g) of *W. koreana*. Shin et al. [24] studied the total phenolic content in different organs of wasabi grown in an organic system. They showed that the total phenolic values in the methanol extract or in the boiled water extract of the roots ranged from 5.10 to 7.78 mg of tannic acid equivalent (TAE)/g of the dry weight (dw.) and from 3.40 to 4.64 mg TAE/g dw., respectively. In turn, the methanol extracts of the flowers and leaves possessed phenolic contents equal to 36.44 and 32.01 mg TAE/g dw., respectively. Thus, the results obtained in our study indicated that *E. japonicum* is a rich source of phenolic compounds. 

The total flavonoid content of the different parts of *E. japonicum* cultivated in Poland was estimated by the previously described colorimetric method [25]. The data were expressed as quercetin equivalents (QE) per g of dry extracts (DE). The results presented in Table 1 showed that, among the examined samples, the flowers (WJA) exhibited the highest content of total flavonoids (45.08 ± 0.18 mg QE/g DE). A comparable content was noted for annual (WJC) and biennial (WJD) leaves (28.81 ± 0.11 and 29.77 ± 0.04 mg QE/g DE, respectively). The data obtained for the roots was five times lower compared to the value noted for the flowers (9.15 ± 0.10 mg QE/g DE). The results obtained in our study for the flowers and leaves were higher than those found by Shin et al. [24]. In their study, a quantitative estimation revealed that a methanol extract from the flowers of *W. japonica* (grown in an organic system) possessed a 11.52 mg QE/g dw. flavonoid content, followed by the leaves—3.25 mg QE/g dw. and fruits—0.64 mg QE/g dw. In the roots, petioles, and floral stalks, the flavonoid content was not detected. The higher amounts of flavonoids were found in the different extracts from the leaves of *W. koreana* (30.16 ± 0.37 to 104.16 ± 8.93 mg NE/g [22]. 

The total phenolic acids content (TPAC) in the studied extracts were presented in Table 1. The amounts ranged from 4.34 ± 0.08 to 17.20 ± 0.24 mg CAE/g DE. The highest TPAC content was observed for the flowers (WJA).

In the next step of our study, the phenolic acid and flavonoid compositions of the extracts obtained from *E. japonicum* were investigated using the LC-MS/MS method. The analysis was carried out using a previously validated and described method [26]. The results of the qualitative and quantitative analyses are presented in Table 2. The sample LC-ESI-MS/MS chromatogram (WJA) and mass spectra of the main compound isosaponarin are displayed in Figures S1 and S2, respectively. 

Among the investigated parts of *E. japonicum*, the flowers (WJA) had the highest total content of phenolic acids and flavonoids. Only six phenolic acids (salicylic acid, *p*-coumaric acid, caffeic acid, ferulic acid, chlorogenic acid, and *cis*-sinapic acid) were identified in all the samples. *Cis*-sinapic acid was the most abundant phenolic acid in all the wasabi extracts (0.09 ± 0.0 to 0.77 ± 0.06 mg per g of DE). Chlorogenic acid was detected in a quantifiable amount in the extracts from the flowers (0.01 ± 0.01 mg/g DE), roots (0.62 ± 0.02 mg/g DE), and biennial leaves (0.42 ± 0.02 mg/g DE). 

The flavonoid aglycones (naringenin, apigenin, kaempferol, eriodyctiol, quercetin, taxifolin, isorhamnetin, and luteolin) were mainly observed in the flower extract. However, their amounts were below the limit of quantification (LOQ). Luteolin was observed in a quantifiable amount (0.01 ± 0.00 mg/g DE) only in the extract from the annual leaves (WJC).

Among the obtained extracts, that of the flowers of *E. japonicum* had the highest total flavonoid glycoside content (17.15 mg/g DE). Quercetin 3-*O*-rutinoside and isovitexin 4’-*O*-glucoside were determined in all the studied parts. The isovitexin 4’-*O*-glucoside content was the highest in the annual leaves and flowers extracts (6.22 ± 0.04 and 6.02 ± 0.10 mg/g DE, respectively). In the flowers extract, great amounts of luteolin 3′,7′-diglucoside and kaempferol 3-*O*-rutinoside were also observed (4.98 ± 0.23 and 2.50 ± 0.06 mg/g DE, respectively). 

According to the literature data, there have not been many reports focusing on the identification of phenolic acids and flavonoids in *E. japonicum*. However, ten flavonoids (namely, isovitexin, isosaponarin, apigenin, luteolin, isoorientin, 7-*O*-*trans*-sinapoylisovitexin, 6″-*O*-(2-*O-trans*-sinapoyl-β-D-glucopyranosyl)-7-*O-trans*-sinapoylisovitexin 4′-*O*-β-D-glucopyranoside, 7-*O*-*trans*-sinapoylisovitexin 4′-*O*-β-D-glucopyranoside, 7-*O-trans*-sinapoylisovitexin 4′-*O*-(6-*O*-*trans*-sinapoyl-β-D-glucopyranoside), and 6′’-*O*-(2-*O-trans*-sinapoyl-β-D-glucopyranosyl)-7-*O*-*trans*-sinapoylisovitexin) were isolated from the fresh leaves of wasabi [9]. Using the HPLC method, Kurata et al. [10] identified luteolin, isorhamnetin-3-glucoside, astragalin, isovitexin, isoorientin, and rutin in the flowers of *W. japonica*. Moreover, from the leaves of *W. japonica* cultivated in Japan, isosaponarin [12,20] and isoorientin were isolated [12]. Moreover, Hosoya et al. [11] isolated *trans*-*p*-hydroxycinnamic acid, *trans*-ferulic acid, *trans*-sinapic acid, 3,4-dimethoxy-*trans*-cinnamic acid, *trans*-ferulic acid methyl ester, *trans*-sinapic acid methyl ester, 3,4-dihydroxy-5-methoxy-*trans*-cinnamic acid, and 3,4-dihydroxy-5-methoxy-*trans*-cinnamic acid methyl ester from the leaves of *W. japonica*. From the roots of *W. japonica* collected in the Republic of Korea, *trans*-*p*-coumaric acid, *trans*-ferulic acid, benzoic acid, and syringic acid were also isolated [27].

Therefore, our results were in good agreement with previous investigations on the identification of isosaponarin and ferulic acid in the leaves [11,20], isorhamnetin-3-glucoside and astragalin in the flowers [10], and *trans*-ferulic acid, as well as *trans*-p-coumaric acid, in the roots [27] of *E. japonicum*. To the best of our knowledge, the other phenolic acids and flavonoids were identified for the first time in the investigated species.

### 2.2. Skin-Related Activities

Skin aging is a natural, unavoidable, complex process caused by oxidative stress. As a result, an increase in the activation of extracellular matrix disruption enzymes and DNA damage was observed. Various intrinsic and extrinsic factors are responsible for this process, including genetic, hormonal, and metabolic changes, as well as exposure to environmental stress [28,29]. As various enzymes such as elastase, collagenase, or hyaluronidase are involved in the skin-aging process, plant extracts, as their inhibitors, are desirable. Thus, apart from the qualitative and quantitative analyses of flavonoids and phenolic acids, this study focused on skin-related activities of different parts of *E. japonicum* cultivated in Poland. In our research, we examined the in vitro antioxidant, anti-collagenase, anti-elastase, anti-hyaluronidase, antibacterial, and cytotoxic activities of different parts of *E. japonicum*.

#### 2.2.1. Antioxidant Activity

The antioxidant activity was studied on a microplate scale in the cell-free systems. All the samples were studied in a concentration range from 10 to 150 μg/mL. It was demonstrated that all the investigated extracts exhibited the moderate scavenging capacity in a concentration-dependent manner (Table 3). For comparison, the radical scavenging activity of ascorbic acid (AA) was tested in the same conditions (IC_50_ = 4.92 ± 0.32 μg/mL). The highest DPPH scavenging activity was shown for the extract from the flowers (WJA) (IC_50_ = 28.72 ± 0.24 μg/mL), followed by annual (WJC) and biennial (WJD) leaves (IC_50_ = 62.84 ± 0.08 and 69.10 ± 0.16 μg/mL, respectively).

The results of other published reports seem to be difficult to compare due to the other conditions used during the experiments. Nevertheless, antioxidant activity using the DDPH assay was studied for different fractions of 80% ethanol extract of the leaves of *W. koreana* [22]. The authors found that extracts of wasabi possessed low scavenging effects, with an IC_50_ value ranging from 522.06 to 12,667.20 μg/mL (the IC_50_ value for the positive control—ascorbic acid—was 41.93 μg/mL). Kim et al. [23] also studied the antioxidant capacity with a DPPH test of different parts of *W. japonica*. They found that the leaves and roots had IC_50_ values of 7.64 ± 0.54 μg/mL and 16.95 ± 0.61 μg/mL, respectively. Eleven compounds isolated from the leaves of *W. japonica* were also evaluated by the DPPH radical scavenging assay. Ferulic acid, luteolin, isoorientin, and rutin showed a significant inhibitory activity. Importantly, luteolin (IC_50_ = 4.09 ± 0.28 μg/mL) and isoorientin (IC_50_ = 6.73 ± 0.45 μg/mL) showed a stronger activity compared to Trolox (IC_50_ = 6.48 ± 0.73 μg/mL) [10].

Similar to the DPPH test, the ABTS^●+^ assay revealed that the extract from the flowers (WJA) possessed the strongest ability to scavenge free radicals (IC_50_ = 11.68 ± 0.47 μg/mL), followed by WJD (IC_50_ = 28.53 ± 0.72 μg/mL) and WJC (IC_50_ = 33.25 ± 0.31 μg/mL). The ABTS^●+^ assay was also used to test the different extracts of *W. koreana*. Kang and coauthors found that different extracts of *W. koreana* possessed a low ABTS radical scavenging effect, with IC_50_ values that ranged from 171.99 to 3103.04 μg/mL (the IC_50_ for the positive control, ascorbic acid, was 39.07 μg/mL) [22]. 

It is commonly known that phenolic compounds may reduce oxidative stress by various mechanisms that depend on their chemical structures. One of them is the chelation of metal ions, such as iron, which plays a key role in the production of harmful oxygen species [30]. Under regular conditions, iron is stored and transported by ferritin or transferrin, which prevents the reaction of free iron ions with reactive oxygen species. The iron ions generate OH• radicals, which can react with lipids, causing their peroxidation [31]. Therefore, it is crucial to search for new natural compounds with the potential ability to chelate metal ions. 

The chelating capacity was determined based on measurement of the percentage of inhibition of the formation of a ferrozine–Fe^2+^ complex. The extracts from the flowers (WJA) and biennial (WJD) and annual (WJC) leaves possessed the activity of interfering with the formation of iron and ferrozine complexes, which suggests their high chelating capacity and ability to capture iron ions before ferrozine. The IC_50_ values for these extracts (12.50 ± 0.36, 13.65 ± 0.29, and 14.27 ± 0.21 µg/mL, respectively) were comparable with that of the positive control—Na_2_EDTA*2H_2_O (IC_50_ = 8.75 ± 0.15 µg/mL). Shin and coauthors also evaluated the metal chelating capacity of different parts of *W. japonica*. They found that the highest chelating activity was expressed by a boiled water extract of the leaves (76.9%), followed by fruits (68.9%), flowers (62.9%), and floral stalks (38.5%) [24]. 

The radical scavenging activities of the phenylpropanoid derivatives isolated from the leaves of *W. japonica* were also evaluated against superoxide anion radicals using an electron spin resonance (ESR) method. The results of this study showed that 1-(3″,4″-dihydroxy-5″-methoxy)-*O*-*trans*-cinnamoyl-2′-*O*-*trans*-sinapoyl gentiobiose, 1-*O*-*trans*-caffeoyl-2′-*O*-*trans*-sinapoyl gentiobiose, 1,2′-di-(3″,4″-dihydroxy-5″-methoxy)-*O*-*trans*-cinnamoyl gentiobiose, 1-(3″,4″-dihydroxy-5′′-methoxy)-*O*-*trans*-cinnamoyl-2′-*O*-*trans*-feruloyl gentiobiose, 3,4-dihydroxy-5-methoxy-*trans*-cinnamic acid, and 3,4-dihydroxy-5-methoxy-*trans*-cinnamic acid methyl ester exhibited IC_50_ values close to 29.0, 85.0, 8.0, 17.0, 36.0, and 31.0 lM, respectively. It is worth underlining that these values were significantly lower compared to the result obtained for the positive control—ascorbic acid (IC_50_ = 140 lM) [11].

#### 2.2.2. Anti-Collagenase Activity 

Collagen, a dominant constituent of normal human dermis that is mainly responsible for its structural stability. Its reduction is started by collagenases, which split interstitial collagens [32]. The inhibition of this enzyme activity can decrease the collagen degradation and, thus, delay wrinkle formation in aging skin. The anti-collagenase effect of the extracts from the flowers, roots, and leaves of *E. japonicum* was measured using *C. histolyticum* collagenase. The results are presented in Table 4.

All the investigated extracts of *E. japonicum* inhibited more than 70% of the collagenase activity. The extract from the flowers (93.34% ± 0.77%) and from the biennial leaves (90.16% ± 0.51%) possessed a significantly higher activity compared to the positive control—EGCG (88.49% ± 0.45%). 

Numerous studies have been demonstrated that flavonoids and phenolic acids are mostly the compounds responsible for the inhibition of collagenase [33,34]. 

#### 2.2.3. Anti-Elastase Activity 

In normal adult skin, the elastin dominates, representing over 90% of the total content of the developed elastic fiber. This protein is primarily responsible for the elasticity of skin [32]. Since there are many reports showing that skin aging is directly related to the breakdown of elastin by the enzyme elastase [33], the elastase inhibitory activity was also determined for the *E. japonicum* extracts.

The analysis was achieved using a N-Succinyl-Ala-Ala-Ala-p-nitroanilide (substrate molecule) and elastase obtained from a porcine pancreas (enzyme). EGCG was used as a positive control, and it inhibited 91.03% ± 0.18% of the enzyme activity. All the extracts showed similar properties, but the highest activity was observed for the extract from the roots—WJB (90.18% ± 0.54%). All the results of the anti-elastase activity are presented in Table 4.

#### 2.2.4. Anti-Hyaluronidase Activity 

Hyaluronic acid, which is located at the periphery of the collagen and elastin fibers in young skin, disappears in aged skin. The decreases in the hyaluronic acid level, caused by an increase in the hyaluronidase activity, can result in changes in the aged skin, such as a reduced turgidity, wrinkling, and altered elasticity. Thus, hyaluronidase inhibitors are useful ingredients, as they have antiaging effects on the skin [35,36]. 

In our study, the extract from the flowers (WJA) exhibited the best hyaluronidase inhibition activity (47.32% ± 0.53%) compared to other samples. Most importantly, such activity was comparable with that of the positive standard—EGCG (62.90% ± 0.12%). In turn, the hyaluronidase inhibition activity for the extracts from the roots (WJB) and annual and biennial leaves (WJC and WJD) was 13.46% ± 0.22%, 28.95% ± 0.69%, and 25.78% ± 0.18%, respectively. 

To the best of our knowledge, previous studies have not reported the in vitro anti-collagenase, anti-elastase, and anti-hyaluronidase activities of *E. japonicum*. Although the influence of isosaponarin isolated from the leaves of *W. japonica* on collagen synthesis in human fibroblasts was investigated. The authors found that this flavone glycoside increased the type I collagen production at the mRNA gene level [20].

#### 2.2.5. Antibacterial Activity

All the samples (extracts and standards) were preliminary tested for their antibacterial activity by the modified disc diffusion method, determining the zones of bacterial growth inhibition. The size of a bacterial growth inhibition zone is directly proportional to the degree of sensitivity of the bacteria against the tested drug. The larger the inhibition zone, the higher the sensitivity of the bacteria against the analyzed agent.

The data indicated that only two studied extracts had strong inhibition activity against Gram-positive, Gram-negative, and aerobic or microaerobic bacterial strains. Among the four *E. japonicum* extracts, only WJB and WJA possessed antibacterial activity (Table 5). This activity was especially focused on the microaerobic strains: *S. mutans*, *S. sanguinis*, *P. acnes* PCM 2400, and *P. acnes* PCM 2334, with the zones of growth inhibition ranging from 16 to 21 mm. Such a narrow spectrum of antimicrobial activity makes it possible to target the therapeutic use of the WJA and WJB extracts against anaerobic pathogens. In turn, two other extracts (WJC and WJD) showed low or no activity against the tested bacterial strains.

In turn, the phenolic acids and flavonoid standards used in our experiment (quercetin—K1; luteolin—K2; gallic acid—K3; *p*-coumaric acid—K4; caffeic acid—K5; vanillic acid—K6), except for K3, had no activity against the tested bacterial strains. K3 showed a broad spectrum of activity against all the strains, ranging from 10 mm to 36 mm.

Moreover, the MIC (minimum inhibitory concentration) of the samples was determined for active extracts, which exhibited the bacterial growth inhibition zones. In the MIC test (Table 6), the activity of the *E. japonicum* extracts was determined in the range of 250–500 μg/mL against the anaerobic strains. The remaining sample determinations against the combination of microorganisms showed no significant activity. Additionally, to distinguish the inhibiting and killing abilities of the tested extracts, the MBC (minimal bactericidal concentration) was determined.

The MIC is the lowest concentration of drug that inhibits the bacterial growth, observed as no turbidity in the culture media. Additionally, the MBC is the lowest concentration that kills the bacteria. It is well-known that bacteriostatic antimicrobial agents have MBC/MIC ratios greater than or equal to 16, while bactericidal antimicrobial agents possess MBC/MIC ratios less than or equal to 4 [37]. In this study, the MBC/MIC ratios for the tested strains (Table 6) indicated that the studied extracts (WJA, WJC, and K3) exhibited a bacteriostatic mode of action against the tested pathogens.

No previous studies have been reported about the antibacterial activity of *E. japonicum* against the bacterial strains tested in this research. However, in a previous study, an antimicrobial protein (WjAMP-1), purified from *W. japonica* leaves, showed an inhibition of growth of *Escherichia coli* (IC_50_ = 8 μg/mL) [38]. Moreover, the roots, stems, and leaves of *W. japonica* collected in Korea and Japan showed bactericidal activities against *H. pylori* strain NCTC 11637 (reference strain), YS 27 (from a duodenal ulcer patient), and YS 50 (from stomach cancer) [39].

#### 2.2.6. Cytotoxic Activity

**Figure 1:**
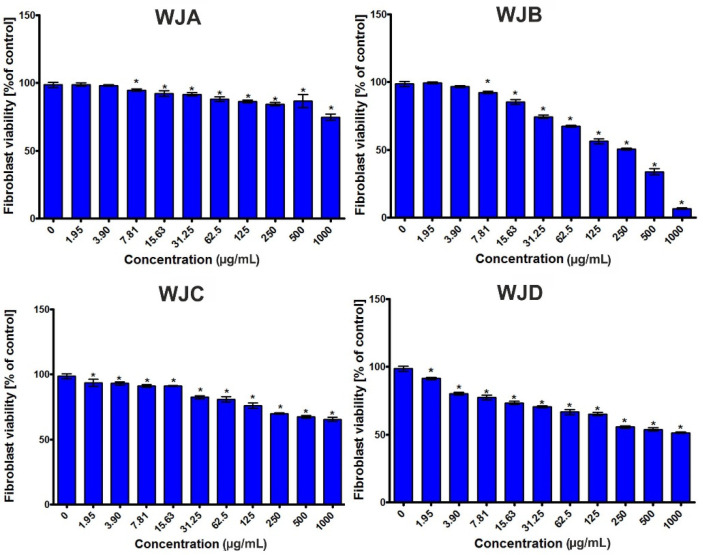
Fibroblast viability (BJ cell line ATTC CRL-2522^TM^) after a 24-h incubation with the WJA, WJB, WJC, and WJD extracts. * Significantly different results compared to the control (culture medium without extracts—0 μg/mL), unpaired *t*-test; *p* < 0.05.

The human skin fibroblast viability after a 24-h incubation with the tested extracts is presented in Figure 1. Taking into account the obtained results, it was observed that the BJ cells showed different responses to the extracts in concentration-dependent manner. In general, most of investigated extracts were nontoxic, with CC_50_ values above 1000 μg/mL (Table 7). Nevertheless, in the case of the WJB extract, it was found that it slightly inhibited the BJ cell viability (Figure 1), with a CC_50_ value close to 254 μg/mL (Table 7). Thus, it was proven that the tested extracts (apart from WJB) were nontoxic towards normal human fibroblasts, and as a consequence, they can be considered very promising substances for further investigations.

In the previous research, the cytotoxic activities of nine compounds isolated from the roots of *W. japonica*, collected in the Republic of Korea, were evaluated against A549 (non-small-cell lung adenocarcinoma), SKOV-3 (ovary malignant ascites), SK-MEL-2 (skin melanoma), and BT549 (invasive ductal carcinoma) cell lines using the sulforhodamine B (SRB) bioassay. In this research, the authors found that *trans*-p-coumaric acid possessed a cytotoxic activity against the BT549 cell line, with an IC_50_ value equal to 10 μM. The other tested compounds (wasabiside A-E, *trans*-ferulic acid, benzoic acid, and syringic acid) were inactive (IC_50_ > 10 μM) against all the tested cancer cell lines used [27].

## 3. Materials and Methods

### 3.1. Chemicals and Reagents 

Ascorbic acid, collagenase from *Clostridium histolyticum*, 2,2-diphenyl-1-picrylhydrazyl radical (DPPH^•^), 2,2′-azino-bis-(3-ethyl-benzothiazole-6-sulfonic acid) (ABTS^●+^), elastase from a porcine pancreas, (-)-epigallocatechin gallate (EGCG), hyaluronidase from the bovine tests, hyaluronic acid sodium salt from a rooster comb, Folin–Ciocalteu reagent, ethylenediaminetetraacetic acid, disodium dihydrate (Na_2_EDTA*2H_2_O), Tricine (≥99%; titration), N-[3-(2-Furyl)acryloyl]-Leu-Gly-Pro-Ala (FALGPA), and N-Succinyl-Ala-Ala-Ala-p-nitroanilide (SANA) were obtained from Sigma-Aldrich (Steinheim, Germany). Phosphate-buffered saline (PBS) was purchased from Gibco (Carlsbad, CA, USA). Reference substances were supplied by ChromaDex (Irvine, CA, USA), while acetonitrile, formic acid, and water for LC analysis were supplied by Merck (Darmstadt, Germany). All other chemicals were of analytical grade and were obtained from the Polish Chemical Reagent Company (POCH, Gliwice, Poland).

### 3.2. Plant Material

The flowers, leaves (annual and biennial), and roots of *Eutrema japonicum* (Miq.) Koidz. were collected from the Wasabi Farm Poland in Jedlnia Letnisko (Poland) at an altitude of 160.3 m a.m.s.l., coordinates 51°43′52″ N, 21°32′27″ E, in January 2021. Taxonomical identification was confirmed by Prof. K. Szewczyk, the botanist from the Department of Pharmaceutical Botany (Medical University in Lublin, Poland). Voucher specimen was deposited in the Department of Pharmaceutical Botany (EJ-250121). 

### 3.3. Preparation of the Extracts

The plant materials were dried in the shade at 24 °C (±0.5 °C) to achieve a constant weight [40]. Extracts were prepared using a mixture of methanol–acetone–water (3:1:1, *v/v/v*; 3 × 100 mL) and were then sonicated at a controlled temperature (40 ± 2 °C) for 30 min [25]. The combined extracts were filtered, concentrated under reduced pressure, and, after freezing, lyophilized in a vacuum concentrator (Free Zone 1 apparatus; Labconco, Kansas City, MO, USA) to obtain dried residues. Dry extracts were weighted and stored in a freezer at −20 °C. The following yields were obtained: flowers (WJA)—1.17 g, roots (WJB)—3.41 g, annual leaves (WJC)—2.41 g, and biennial leaves (WJD)—4.71 g.

### 3.4. Total Flavonoid and Phenolic and Phenolic Acid Contents

Total flavonoid (TFC) and total phenolic contents (TPC) were established using the colorimetric assays, as described previously [25]. The absorbances were measured at 430 and 680 nm, respectively, using a Pro 200F Elisa Reader (Tecan Group Ltd., Männedorf, Switzerland). TPC was estimated from the calibration curve (R2 = 0.9845) using gallic acid as a standard (concentration range 0.002–0.1 mg/mL). The results were expressed as the mg of gallic acid equivalents (GAE) per 1 g of dry extract (DE). TFC was estimated from the calibrated curve (R2 = 0.995) using quercetin (0.004–0.11 mg/mL) as the standard. The results were expressed as the mg of quercetin equivalents (QE) per 1 g of DE. The total phenolic acids (TPAC) content was assessed using Arnov’s reagent, as described in Polish Pharmacopoeia IX (an official translation of PhEur 7.0) [40]. The absorbance was measured at 490 nm. The TPAC was estimated from the calibration curve (R2 = 0.9999) using caffeic acid as a standard in the concentration of 3.36–23.52 μg/mL. The results were expressed as the mg of caffeic acid equivalents (CAE) per 1 g of DE.

### 3.5. LC-ESI-MS/MS Analysis

The Agilent 1200 Series HPLC system (Agilent Technologies, Palo Alto, CA, USA) coupled to a 3200 QTRAP mass spectrometer (AB Sciex, Redwood City, CA, USA) was used for the qualitative and quantitative analyses of flavonoids and phenolic acids in different parts of the *E. japonicum* extracts. The separation of the analyzed compounds, injected in a 3-µL amount, was performed on a Zorbax SB-C18 analytical column (2.1 × 100 mm, 1.8 µm; Agilent Technologies, Palo Alto, CA, USA) at 25 °C. Elution was carried out using solvent A (0.1% HCOOH in water) and solvent B (0.1% HCOOH in acetonitrile). The following gradient elution program was used: 0–2 min—20% B, 3 to 4 min—25% B, 5 to 6 min—35% B, 5 to 6 min—35% B, 8–12 min—65% B, 14–16 min—80% B, and 20–28 min—20% B. The flow rate was 300 µL/min. The mass spectra of the analyzed compounds were acquired in the negative ESI mode, and the optimum values of the source parameters were as follows: capillary temperature 450°C, nebulizer gas 50 psi, curtain gas 30 psi, and source voltage −4500 V for phenolic acids and flavonoid glycosides and capillary temperature 550 °C, nebulizer gas 30 psi, curtain gas 20 psi, and source voltage −4500 V for the flavonoid aglycones analysis. Details of the LC-ESI-MS/MS analysis are presented in Table 8 and Table 9. Analyst 1.5 software (AB Sciex, Redwood City, CA, USA) was used for the analysis and data acquisition. 

### 3.6. Antioxidant Activity 

All antioxidant and enzyme inhibitory assays were done in 96-well plates (Nunclon, Nunc, Roskilde, Denmark) using an Infinite Pro 200F Elisa Reader (Tecan Group Ltd., Männedorf, Switzerland). The experiments were performed in triplicate.

#### 3.6.1. DPPH• Assay

The 2,2-diphenyl-1-picryl-hydrazyl (DPPH•) free radical scavenging activity of the *E. japonicum* extracts and positive control—ascorbic acid (AA)—was studied using the method described previously [25] but with some modifications. After 30 min of incubation at 28 °C, the decrease in DPPH• absorbance, caused by the tested extracts, was measured at 517 nm. The results were expressed as the values of the IC_50_.

#### 3.6.2. ABTS^●+^ Assay

The ABTS^●+^ decolorization assay was the second method applied for an assessment of the antioxidant activity [25]. The absorbance was measured at 734 nm. Trolox was used as a positive control. The results were expressed as the values of the IC_50_.

#### 3.6.3. Metal Chelating Activity (CHEL)

The metal chelating activity was established using the method described by Guo et al. [41], modified in our previous study [25,42]. The absorbance was measured at 562 nm. As a positive control, Na_2_EDTA*2H_2_O was used. The results were expressed as the IC_50_ values of the *E. japonicum* extracts based on the concentration–inhibition curves.

### 3.7. Enzyme Inhibitory Activity

#### 3.7.1. Anti-Elastase Activity 

Anti-elastase activity was measured spectrophotometrically according to Chiocchio et al. [43]. Porcine pancreatic elastase (3.33 mg/mL; 25 µL) and *E. japonicum* samples (100 µg/mL) were incubated in Tris-buffer (0.2 mM, pH 8.0) for 10 min at 29 °C. To start the reaction, N-Succinyl-Ala-Ala-Ala-p-nitroanilide (2 mM; 125 µL) as a substrate was added. After 15 min of incubation, the absorbance was measured at 485 nm. Epigallocatechin gallate (100 µg/mL) was used as a positive control.

#### 3.7.2. Anti-Collagenase Activity 

Anti-collagenase activity was studied using N-[3-(2-furyl) acryloyl]-Leu-Gly-Pro-Ala (FALGPA) as a substrate and was measured according to Mandrone et al. [44]. Collagenase from *Clostridium histolyticum* (20 mU), dissolved in Tricine buffer (pH 7.5, 0.05 M, containing 0.4-M natrium chloride and 0.01-M calcium chloride), and *E. japonicum* samples (100 µg/mL) were incubated for 10 min at 35 °C. To start the reaction, FALGPA (1 mM) was added. After 15 min of incubation at 29 °C, the absorbance was measured at 345 nm. Epigallocatechin gallate (100 µg/mL) was used as a positive control. 

#### 3.7.3. Anti-Hyaluronidase Activity

Anti-hyaluronidase activity was established using the method described by Liyanaarachchi et al. [45]. After 20 min of incubation at 37 °C, the absorbance was measured at 585 nm. Epigallocatechin gallate (100 µg/mL) was used as a positive control. 

### 3.8. Antimicrobial Activity

#### 3.8.1. Bacterial Strains

All the tested extracts were screened for their in vitro antibacterial activity against strains: aerobic Gram-positive *Staphylococcus aureus* ATCC 25923, *Staphylococcus epidermidis* ATCC 12228, aerobic Gram-negative *Escherichia coli* ATCC 25992, *Pseudomonas aeruginosa* ATCC 27853, microaerobic Gram-positive *Propionibacterium acnes* PCM 2400, *Propionibacterium acnes* PCM 2334, *S. mutans* PCM 2502, and *S. sanguinis* PCM 2335. For determination of the antibacterial activity, Mueller–Hinton agar or broth (MH-agar or MH-broth) for aerobic and Brain–Heart Infusion agar or broth (BHI-agar or BHI-broth) for microaerobic strains were used. Bacterial inoculum was prepared by subculturing microorganisms into MH-agar or BHI-agar at 37 °C for 24 h or 48 h, respectively. The growth was harvested using 5 mL of 0.9% NaCl and diluted to 0.5 McFarland (1.5 × 10^8^ CFU/mL (CFU: colony-forming unit)).

#### 3.8.2. Agar Disc Diffusion Assay

The antibacterial character of all the samples was initially determined by a disc diffusion assay [46]. The bacterial inoculum was spread on the agar surface of the Petri plates by applying a cotton swab. The solutions of all the tested samples (100 µg/mL in DMSO) were placed on inoculated Petri plates. The plates with MH-agar (for aerobic strains) were incubated for 24 h at 37 °C, while the plates with BHI-agar were incubated for 48 h at 37 °C. After incubation, the diameter of the growth inhibition zone around the samples was measured.

#### 3.8.3. MIC (Minimum Inhibitory Concentration) and MBC (Minimal Bactericidal Concentration) Analysis 

The minimum inhibitory concentration (MIC) of the extracts was determined for the bacterial strains, which exhibited the bacterial growth inhibition zones. The test was performed using double-serial microdilution in the 96-well microtiter plates, according to the CLSI method but with some modifications [47]. Two hundred microliters of broth were pipetted into each well. The double-serial dilution of the tested derivatives was performed in the test wells, resulting in concentrations that ranged from 2000 µg/mL to 0.098 µg/mL. Finally, 2 µL of tested bacteria inoculum were added to the wells (except for the negative sterility control and blanc dye control). The tests were performed at 37 °C for 24 h (aerobic strains) or for 48 h (microaerobic strains). After incubation, the panel was digitally analyzed at 600 nm using the microplate reader BioTek™ Synergy™ H4 Hybrid Microplate Reader (Thermo Fisher Scientific, Waltham, MA, USA). The growth intensity in the sample well was compared to the negative and positive controls. Additionally, the MBC (minimal bactericidal concentration) was determined by spreading 10 µL of liquid from a clear sample well on the agar medium. The plates were incubated for 24 h at 37 °C, and the MBC was defined as the lowest concentration of sample without bacterial growth. Each experiment was repeated in triplicate.

### 3.9. Cytotoxicity Evaluation

The cytotoxic effect of the tested extracts was assessed in accordance with our procedure described earlier [48,49]. The experiment was carried out using the BJ cell line (normal human skin fibroblasts, ATCC CRL-2522^TM^). Briefly, the BJ cells were seeded on a 96-well plate at a concentration of 1.5 × 10^4^ cells/well and incubated for 24 h at 37 °C in a humidified atmosphere (5% CO_2_, 95% air). On the next day, stock solutions of extracts (100 mg/mL) in dimethyl sulfoxide (DMSO) were obtained, and then, two-fold serial dilutions of extracts (1000–1.95 μg/mL) in the culture medium were prepared. Subsequently, the culture medium was replaced by an appropriate dilution of extracts, and the treated BJ cells were incubated for 24 h at 37 °C, 5% CO_2_, 95% air. Cytotoxicity of the tested extracts was evaluated using the MTT assay [50]. Based on the obtained results, the values of the half-maximum cytotoxic concentration (CC_50_) were determined (4-parameter nonlinear regression analyses, GraphPad Prism 5, version 5.04, Software, GraphPad Software, San Diego, CA, USA). The CC_50_ refers to a concentration of extract required for the reduction of BJ cell viability to 50%.

### 3.10. Statistical Analysis

The results were expressed as the mean values ± standard deviations (SD) of three independent experiments. The data from the cell culture experiments were subjected to a statistical analysis using an unpaired Student’s *t*-test, and the differences were considered significant when *p* < 0.05 (GraphPad Prism 5, version 5.04 Software, GraphPad Software, San Diego, CA, USA).

## 4. Conclusions

In this in vitro study, we characterized the composition and comprehensively evaluated the biological activities of the extracts from the flowers (WJA), roots (WJB), and leaves—annual (WJC), as well as biennial (WJD)—of *Eutrema japonicum* cultivated at Wasabi Farm Poland. Thus, the content of flavonoids and phenolic acids in the extracts was determined, as well as the antioxidant, anti-collagenase, anti-elastase, anti-hyaluronidase, antimicrobial, and cytotoxic properties of such extracts.

Among the tested samples, the WJA extract possessed the most desirable biological activities. It exhibited the greatest antioxidant features, as proven by the DPPH^•^ assay, ABTS^●+^ test, and Metal Chelating Activity test. Moreover, it had the greatest anti-collagenase and anti-hyaluronidase features compared to the other investigated extracts. The determined MIC and MBC values proved that the WJA extract also exhibited the most promising antibacterial activity, as it possessed bacteriostatic activity against the anaerobic pathogens. In turn, the cell culture experiments indicated that the WJA extract was noncytotoxic towards normal skin human fibroblasts. The beneficial biological properties of the WJA extract seemed to be correlated with its composition. We proved that WJA is rich in flavonoids, especially isovitexin 4’-*O*-glucoside, luteolin 3′,7′-diglucoside, and kaempferol 3-*O*-rutinoside. It is worth underlining that the aforementioned properties revealed in this study allowed us to suppose that the WJA extract has “multidirectional” activity. Thus, considering a broad spectrum of activities of such an extract, it seems to be a promising antiaging agent. For this reason, the WJA extract will be subjected to further in vivo studying in order to confirm its beneficial activity.

## Figures and Tables

**Table 1:** The total content of phenolic (TPC), flavonoid (TFC), and phenolic acids (TPAC) in the flowers (WJA), roots (WJB), and leaves (annual—WJC and biennial—WJD) of *E. japonicum*. DE—dry extract; GAE—Gallic Acid Equivalent; CAE—Caffeic Acid Equivalent; QE—Quercetin Equivalent. Values were presented as the mean ± standard deviation (*n* = 9).

Sample	Total Phenolic Content (mg GAE/g DE)	Total Phenolic Acids (mg CAE/g DE)	Total Flavonoid Content (mg QE/g DE)
WJA	187.79 ± 6.86	17.20 ± 0.24	45.08 ± 0.18
WJB	31.55 ± 2.17	4.34 ± 0.08	9.15 ± 0.10
WJC	77.92 ± 4.82	13.84 ± 0.09	28.81 ± 0.11
WJD	54.93 ± 0.99	9.70 ± 0.20	29.77 ± 0.04

**Table 2:** Content of phenolic acids and flavonoids in the leaves, roots, and flowers of *E. japonicum*. nd—not detected; LOQ—limit of quantification; DE—dry extract.

No	Compound	Amounts (mg/g DE)			
		WJA	WJB	WJC	WJD
1	salicylic acid	<LOQ	<LOQ	<LOQ	nd
2	*p*-coumaric acid	0.10 ± 0.02	nd	0.01 ± 0.01	<LOQ
3	caffeic acid	<LOQ	nd	<LOQ	<LOQ
4	ferulic acid	<LOQ	nd	1.21 ± 0.07	<LOQ
5	chlorogenic acid	0.01 ± 0.01	0.62 ± 0.02	nd	0.42 ± 0.02
6	*cis*-sinapic acid	0.67 ± 0.07	0.09 ± 0.00	0.77 ± 0.06	0.56 ± 0.01
7	apigenin	<LOQ	nd	<LOQ	<LOQ
8	naringenin	<LOQ	nd	nd	nd
9	kaempferol	<LOQ	nd	nd	nd
10	eriodictiol	<LOQ	nd	nd	nd
11	quercetin	<LOQ	nd	nd	nd
12	taxifolin	<LOQ	nd	nd	nd
13	isorhamnetin	<LOQ	nd	nd	nd
14	luteolin	<LOQ	nd	0.01 ± 0.00	<LOQ
15	apigenin 7-*O*-glucoside	0.02 ± 85.0	nd	<LOQ	nd
16	naringenin 7-*O*-glucoside	<LOQ	nd	nd	nd
17	apigenin 6-*C*-glucoside/apigenin 8-*C*-glucoside	0.52 ± 0.07	nd	2.57 ± 0.01	0.70 ± 0.09
18	luteolin 7-*O*-glucoside	0.44 ± 0.12	nd	0.18 ± 0.00	0.13 ± 0.02
19	kaempferol 3-*O*-glucoside	0.38 ± 0.01	nd	<LOQ	nd
20	quercetin 3-*O*-glucoside	0.53 ± 0.07	nd	<LOQ	<LOQ
21	isorhamnetin 3-*O*-glucoside	0.48 ± 0.02	nd	nd	nd
22	kaempferol 3-*O*-rutinoside	2.50 ± 0.06	nd	<LOQ	nd
23	quercetin 3-*O*-rutinoside	0.74 ± 0.03	<LOQ	<LOQ	<LOQ
24	luteolin 3′,7′-diglucoside	4.98 ± 0.23	nd	3.51 ± 0.13	2.74 ± 0.10
25	isorhamnetin 3-*O*-rutinoside	0.54 ± 0.04	nd	nd	nd
26	isovitexin 4’-*O*-glucoside	6.02 ± 0.10	<LOQ	6.22 ± 0.04	5.99 ± 0.22

**Table 3:** The IC_50_ values determined in the antioxidant tests. Data were expressed as the mean ± SD, *n* = 3. AA—ascorbic acid; Na_2_EDTA*2H_2_O—ethylenediaminetetraacetic acid, disodium dihydrate; nt—not tested. The flowers (WJA), roots (WJB), and leaves (annual—WJC and biennial—WJD) of *E. japonicum*. DPPH—2,2-diphenyl-1-picryl-hydrazyl radical; ABTS—2,2′-azino-bis-(3-ethyl-benzothiazole-6-sulfonic acid; CHEL—metal chelating activity. * Statistically significant differences compared to AA; # statistically significant differences compared to Na_2_EDTA*2H_2_O, *p* < 0.05.

Sample	IC_50_		
	DPPH (μg/mL)	ABTS (μg/mL)	CHEL (μg/mL)
WJA	28.72 ± 0.24 *	11.68 ± 0.47	12.50 ± 0.36 #
WJB	134.30 ± 1.75 *	65.18 ± 0.73	32.08 ± 0.13 #
WJC	62.84 ± 0.08 *	33.25 ± 0.31	14.27 ± 0.21 #
WJD	69.10 ± 0.16 *	28.53 ± 0.72	13.65 ± 0.29 #
AA	4.92 ± 0.32	nt	nt
Trolox	nt	3.14 ± 0.17	nt
Na_2_EDTA*2H_2_O	nt	nt	8.75 ± 0.15

**Table 4:** Anti-collagenase, anti-elastase, and anti-hyaluronidase activities of the flowers (WJA), roots (WJB), and leaves (annual—WJC and biennial—WJD) of *E. japonicum*. * Statistically significant differences compared to epigallocatechin gallate (EGCG), *p* < 0.05.

Sample	Inhibition (%)		
	Collagenase Inhibition	Elastase Inhibition	Hyaluronidase Inhibition
WJA	93.34 ± 0.77 *	88.93 ± 0.16 *	47.32 ± 0.53 *
WJB	78.42 ± 0.25 *	90.18 ± 0.54 *	13.46 ± 0.22 *
WJC	87.51 ± 0.83 *	84.90 ± 0.60	28.95 ± 0.69 *
WJD	90.16 ± 0.51 *	88.89 ± 0.36 *	25.78 ± 0.18 *
EGCG	88.49 ± 0.45	91.03 ± 0.18	62.90 ± 0.12

**Table 5:** Zones of bacterial growth inhibition expressing the antibacterial activity of the *E. japonicum* extracts. Extracts from the flowers (WJA), roots (WJB), and leaves (annual—WJC and biennial—WJD) of *E. japonicum*. K1—quercetin; K2—luteolin; K3—gallic acid; K4—*p*-coumaric acid; K5—caffeic acid; K6—vanillic acid.

Bacterial Strains	Zones of Bacterial Growth Inhibition (mm)									
	WJA	WJB	WJC	WJD	K1	K2	K3	K4	K5	K6
*S. aureus* ATCC 25923	0	0	0	0	0	0	15	0	0	0
*S. epidermidis* ATCC 12228	0	0	0	0	0	0	36	0	28	0
*E. coli ATCC 25992*	7	6	0	0	0	0	12	8	4	4
*P. aeruginosa ATCC 27853*	0	0	0	0	0	0	10	6	4	4
*S. mutans PCM 2502*	16.5	16	0	0	0	0	16	0	0	0
*P. acnes PCM 2400*	16	19.5	15	8	0	0	20	0	0	0
*P. acnes PCM 2334*	17	21	16	10	4	0	22	0	0	0
*S. sanguinis PCM 2335*	17	18	0	0	0	0	18	0	0	0

**Table 6:** Minimum inhibitory concentration (MIC (μg/mL)) and MBC/MIC ratio as the antibacterial activity of the *E. japonicum* extracts.

Bacterial Strains	Sample		
	WJA	WJB	K3
*S. aureus* ATCC 25923	-	-	125
MBC/MIC			16
*S. epidermidis* ATCC 12228	-	-	31.25
MBC/MIC	-	-	8
*E. coli ATCC 25992*	1000	1000	500
MBC/MIC	-	-	8
*P. aeruginosa ATCC 27853*	-	-	1000
MBC/MIC	-	-	-
*S. mutans PCM 2502*	500	500	125
MBC/MIC	-	8	8
*P. acnes PCM 2400*	500	250	62.5
MBC/MIC	8	8	8
*P. acnes PCM 2334*	250	125	62.5
MBC/MIC	8	16	8
*S. sanguinis PCM 2335*	500	250	62.5
MBC/MIC	-	8	8

**Table 7:** The half-maximal cytotoxic concentrations (CC_50_) of the tested extracts. The CC_50_ values were determined against normal human skin fibroblasts (BJ cell line ATTC CRL-2522TM) after a 24-h incubation.

Extract	CC_50_ (μg/mL)
WJA	>1000
WJB	245.45
WJC	>1000
WJD	>1000

**Table 8:** LC-ESI-MS/MS analytical results of the phenolic acids and flavonoids.

Compound	Retention Time (min)	Q1/Q3 (*m/z*)	DP (V)	EP (V)	CEP (V)	CE (eV)	CXP (V)
Phenolic acids							
5-caffeoylquinic acid (chlorogenic acid)	9.30	352.9/190.8	−35	−4.5	−16	−24	−2
	10.42	352.9/84.9	−35	−4.5	−16	−60	0
Caffeic acid	11.38	178.7/88.9	−30	−6.5	−12	−46	0
		178.7/134.9	−30	−6.5	−12	−16	0
4-Hydroxycinnamic acid (*p*-coumaric acid)	14.10	162.7/119	−30	−8	−12	−14	0
		162.7/93	−30	−8	−12	−44	0
Sinapic acid	14.47	222.8/121	−35	−8.5	−10	−36	0
	14.94	222.8/148.9	−35	−8.5	−10	−20	0
Ferulic acid	14.80	192.8/133.9	−25	−11.5	−14	−16	0
	15.22	192.8/177.9	−25	−11.5	−14	−12	−2
Salicylic acid	17.91	136.8/93	−35	−4	−10	−16	−2
		136.8/75	−35	−4	−10	−48	0
Flavonoid aglycones							
Naringenin	14.52	270.8/119	−50	−11.5	−12	−34	0
		270.8/150.9	−50	−11.5	−12	−22	0
Taxifolin	15.15	302.7/124.9	−45	−3.5	−18	−26	0
		302.7/284.8	−45	−3.5	−18	−14	−4
Luteolin	17.82	284.7/132.9	−75	−9	−18	−38	0
		284.7/150.9	−75	−9	−18	−26	0
Eriodyctiol	17.89	286.7/134.9	−45	−6	−12	−32	0
		286.7/150.9	−45	−6	−12	−18	−2
Quercetin	17.94	300.7/150.9	−60	−2.5	−12	−26	0
		300.7/178.8	−60	−2.5	−12	−20	−2
Apigenin	18.64	268.8/117	−70	−9.5	−12	−44	0
		268.8/106.8	−70	−9.5	−12	−34	0
Kaempferol	18.85	284.7/116.8	−70	−5	−12	−46	0
		284.7/93	−70	−5	−12	−52	0
Isorhamnetin	18.99	314.7/299.7	−65	−2.5	−26	−20	−4
		314.7/150.9	−65	−2.5	−26	−30	0
Flavonoid glycosides							
Isovitexin 4’-*O*-glucoside (Isosaponarin)	8.82	593.2/282.1	−25	−10	−34	−30	−3
		593.2/473.1	−25	−10	−34	−30	−3
Luteolin 3′,7′-diglucoside	11.28	609.1/285	−70	−7.5	−28	−50	−4
		609.1/447	−70	−7.5	−28	−32	−18
Quercetin 3-*O*-rutinoside (Rutin)	11.99	608.7/299.6	−90	−8	−30	−46	−4
		608.7/270.9	−90	−8	−30	−60	−4
Apigenin 6-*C*-glucoside (Isovitexin)	12.38	430.8/310.9	−65	−4.5	−18	−28	−4
		430.8/340.9	−65	−4.5	−18	−26	−14
Apigenin 8-*C*-glucoside (Vitexin)	12.40	430.8/310.9	−75	−4.5	−20	−26	−4
		430.8/340.9	−75	−4.5	−20	−34	−14
Luteolin 7-*O*-glucoside (Luteoloside)	12.87	446.8/284.8	−70	−10.5	−20	−30	−4
		446.8/132.9	−70	−10.5	−20	−78	0
Quercetin 3-*O*-glucoside (Isoquercetin)	13.00	462.7/299.7	−85	−1.5	−20	−30	−4
		462.7/270.7	−85	−1.5	−20	−44	−4
Kaempferol 3-*O*-rutinoside (Nicotiflorin)	13.31	592.7/284.8	−65	−12	−30	−38	−2
		592.7/226.7	−65	−12	−30	−68	−2
Isorhamnetin 3-*O*-rutinoside (Narcissoside)	13.52	622.8/314.9	−90	−4.5	−30	−40	−4
		622.8/298.8	−90	−4.5	−30	−52	−4
Kaempferol 3-*O*-glucoside (Astragalin)	14.66	446.7/226.8	−75	−9	−20	−54	−2
		446.7/254.8	−75	−9	−20	−40	−2
Isorhamnetin 3-*O*-glucoside	14.76	476.8/313.9	−95	−10	−22	−30	−4
		476.8/270.9	−95	−10	−22	−44	−4
Apigenin 7-*O*-glucoside (Apigetrin and Cosmosiin)	14.91	430.7/267.7	−70	−9	−20	−38	−4
		430.7/116.9	−70	−9	−20	−84	0
Naringenin 7-*O*-glucoside	15.12	432.7/270.8	−40	−8.5	−20	−22	−4
		432.7/118.9	−40	−8.5	−20	−64	0

**Table 9:** Analytical results of the LC-ESI-MS/MS quantitative method of the phenolic acids and flavonoids. Limit of quantification (LOQ), limit of detection (LOD), and calibration curve parameters.

Compound	LOD (ng/mL)	LOQ (ng/mL)	R^2^	Linearity Range (ng/mL)
Phenolic acids				
5-Caffeoylquinic acid (chlorogenic acid)	70	150	0.9989	150–15,000
Caffeic acid	200	400	0.9991	400–20,000
4-Hydroxycinnamic acid (*p*-coumaric acid)	50	200	0.9990	500–15,000
Sinapic acid	500	1000	0.9989	1000–30,000
Ferulic acid	1000	1500	0.9985	1830–35,000
Salicylic acid	300	500	0.9974	1500–15,000
Flavonoid aglycones				
Naringenin	150	300	0.9982	600–6000
Taxifolin	10	20	0.9984	40–10,000
Luteolin	5	15	0.9987	50–1500
Eriodyctiol	25	50	0.9985	100–6600
Quercetin	50	100	0.9982	100–6600
Apigenin	15	22	0.9970	100–4000
Kaempferol	30	50	0.9971	150–3000
Isorhamnetin	300	500	0.9990	1000–5000
Flavonoid glycosides				
Isovitexin 4’-*O*-glucoside (Isosaponarin)	100	250	0.9958	500–10,000
Luteolin 3′,7′-diglucoside	250	500	0.9980	1000–25,000
Quercetin 3-*O*-rutinoside (Rutin)	100	250	0.9980	2000–28,800
Apigenin 6-*C*-glucoside (Isovitexin)	100	250	0.9991	1500–50,000
Apigenin 8-*C*-glucoside (Vitexin)	100	200	0.9981	2000–50,000
Luteolin 7-*O*-glucoside (Luteoloside)	50	100	0.9982	200–25,000
Quercetin 3-*O*-glucoside (Isoquercetin)	100	250	0.9975	2500–50,000
Kaempferol 3-*O*-rutinoside (Nicotiflorin)	60	120	0.9957	150–60,000
Isorhamnetin 3-*O*-rutinoside (Narcissoside)	50	100	0.9981	150–25,000
Kaempferol 3-*O*-glucoside (Astragalin)	100	250	0.9974	1500–25,000
Isorhamnetin 3-*O*-glucoside	100	150	0.9969	2000–35,000
Apigenin 7-*O*-glucoside (Apigetrin, Cosmosiin)	100	250	0.9989	1500–25,000
Naringenin 7-*O*-glucoside	100	200	0.9990	250–25,000

## Data Availability

Data available on request.

## Supplementary Materials

The following are available online at https://www.mdpi.com/article/10.3390/ijms22126219/s1. Figure S1. The chromatogram in the Multiple Reaction Monitoring (MRM) mode of phenolic acids, flavonoid aglycones and glycosides in the WJA extract: 1-chlorogenic acid; 2- *p*-coumaric acid; 3- sinapic acid; 4 – isosaponarin; 5 - luteolin 3’,7’-diglucoside; 6 – rutin; 7 – isovitexin/vitexin; 8 -luteolin-7-*O*-glucoside; 9 - isoquercetin; 10 - kaempferol-3-*O*-rutinoside; 11-narcissoside; 12 - astragalin; 13 - isorhamnetin-3-*O*-glucoside; 14 - apigenin 7-*O*-glucoside. Figure S2. Mass spectra of isosaponarin by ESI in negative mode.
