# Supplementary material for: Flavonoid and Phenolic Acids Content and In Vitro Study of the Potential Anti-Aging Properties of Eutrema japonicum (Miq.) Koidz Cultivated in Wasabi Farm Poland
Source: Int J Mol Sci. 2021 Jun 9;22(12):6219. doi: 10.3390/ijms22126219 (PMC8229664; doi:10.3390/ijms22126219)
Supplement: Supplementary file 1 [file ijms-22-06219-s001.zip › ijms-1246187-supplementary.pdf]

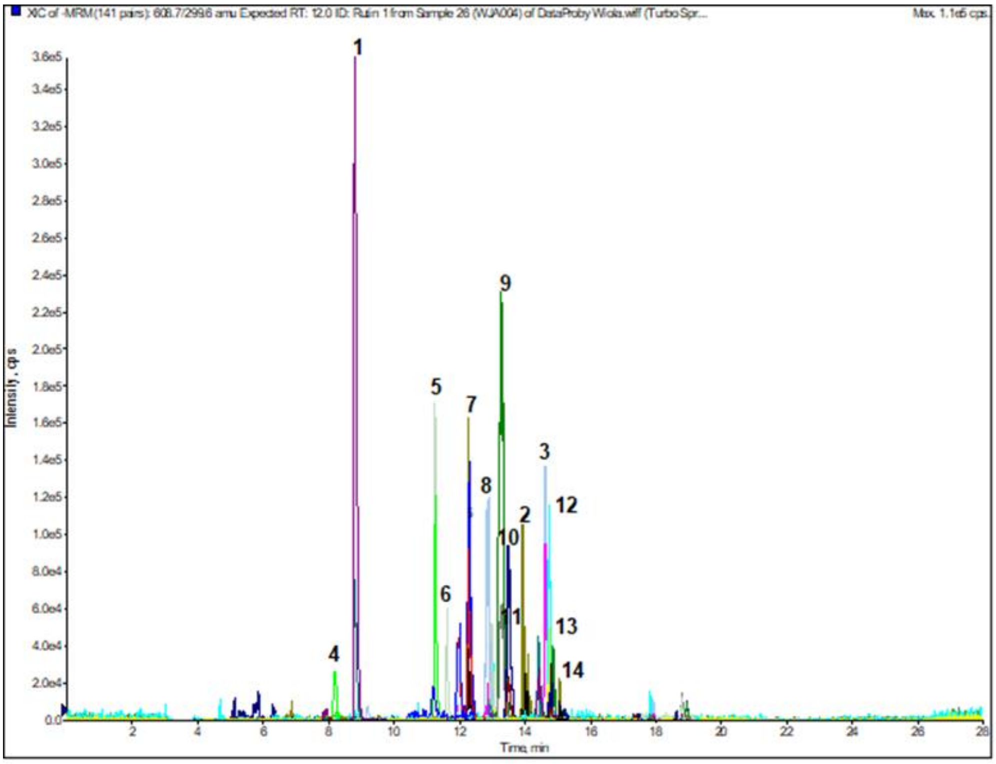

Figure S1. The chromatogram in the Multiple Reaction Monitoring (MRM) mode of phenolic acids, flavonoid aglycones and glycosides in the WJA extract: 1- chlorogenic acid; 2- *p*-coumaric acid; 3- sinapic acid; 4 – isosaponarin; 5 - luteolin 3′,7′-diglucoside; 6 – rutin; 7 – isovitexin/vitexin; 8 -luteolin-7-*O*-glucoside; 9 - isoquercetin; 10 - kaempferol-3-*O*-rutinoside; 11-narcissoside; 12 - astragalin; 13 - isorhamnetin-3-*O*-glucoside; 14 - apigenin 7-*O*-glucoside.

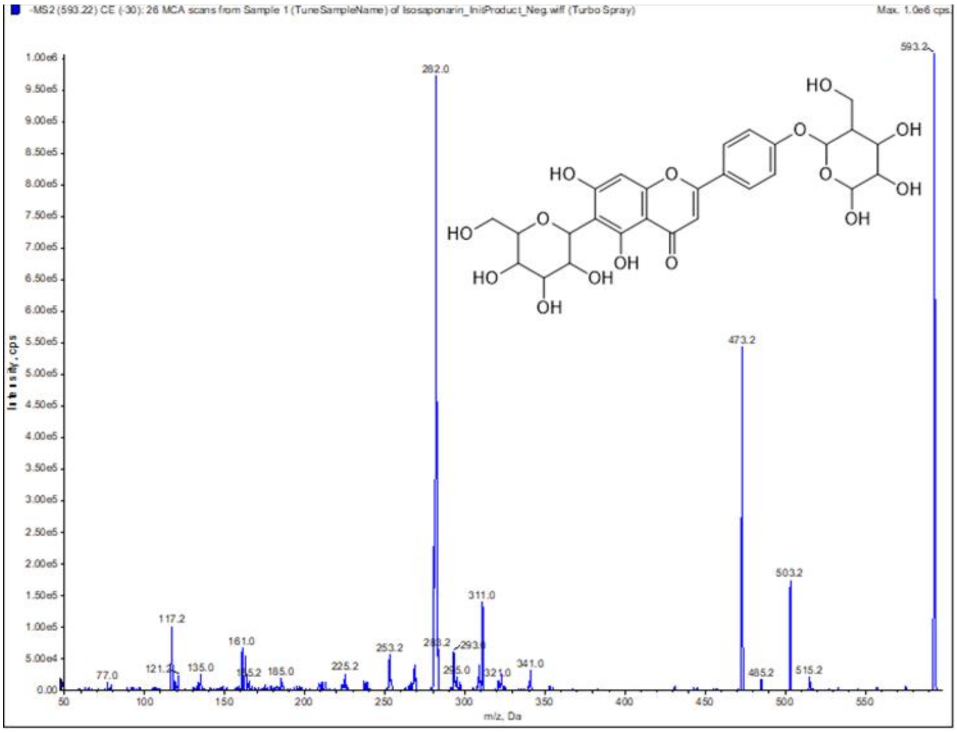

Figure S2. Mass spectra of isosaponarin by ESI in negative mode.
